# Acute lyme infection presenting with amyopathic dermatomyositis and rapidly fatal interstitial pulmonary fibrosis: a case report

**DOI:** 10.1186/1752-1947-4-187

**Published:** 2010-06-21

**Authors:** Hien Nguyen, Connie Le, Hanh Nguyen

**Affiliations:** 1 Internal Medicine Department, Kaiser Permanente, Mid-Atlantic, 6104 Old Branch Avenue, Temple Hills, MD, 20748, USA; 2 Internal Medicine Department, Fairfax Hospital, 3300 Gallows Road, Falls Church, VA, 22042, USA; 3 Family Medicine Department, University of California, Irvine Medical Center, 1001 Health Sciences Road, 252 Irvine Hall, Irvine, CA, 92697, USA

## Abstract

**Introduction:**

Dermatomyositis has been described in the setting of lyme infection in only nine previous case reports. Although lyme disease is known to induce typical clinical findings that are observed in various collagen vascular diseases, to our knowledge, we believe that our case is the first presentation of acute lyme disease associated with amyopathic dermatomyositis, which was then followed by severe and fatal interstitial pulmonary fibrosis only two months later.

**Case presentation:**

We present a case of a 64-year-old African-American man with multiple medical problems who was diagnosed with acute lyme infection after presenting with the pathognomonic rash and confirmatory serology. In spite of appropriate antimicrobial therapy for lyme infection, he developed unexpected amyopathic dermatomyositis and then interstitial lung disease.

**Conclusions:**

This case illustrates a potential for lyme disease to produce clinical syndromes that may be indistinguishable from primary connective tissue diseases. An atypical and sequential presentation (dermatomyositis and interstitial lung disease) of a common disease (lyme infection) is discussed. This case illustrates that in patients who are diagnosed with lyme infection who subsequently develop atypical muscular, respiratory or other systemic complaints, the possibility of severe rheumatological and pulmonary complications should be considered.

## Introduction

Dermatomyositis has been described in the setting of lyme infection in only nine previous case reports. Our case is unique in the rapidity and severity of the subsequent fatal interstitial lung disease (ILD). In spite of a link of dermatomyositis with varied medical conditions, its pathogenesis remains elusive. Lyme disease is known to induce a constellation of clinical symptoms which are characteristic of various collagen vascular diseases. In summary, we describe a novel and highly unusual case in which the diagnosis of lyme infection was followed by amyopathic dermatomyositis and then interstitial pulmonary fibrosis.

## Case presentation

A 64-year-old African-American man with prostate cancer, chronic obstructive pulmonary disease (COPD), and hepatitis C virus (HCV) infection, presented with four weeks of polyarthralgias of bilateral knees, hands, and neck, which were preceded two weeks previously by a 5 cm annular lesion on his abdomen with central clearing consistent with erythema migrans (EM). The lyme IgM antibody testing was positive and consistent with acute *Borrelia burgdoferi *infection, while all antibody bands for *B. burdorferi *IgG were nonreactive. These results were confirmed by western blot, with positive 25 and 41 KD antibody IgM bands, in accordance with Centers for Disease Control's (CDC) diagnostic serologic criteria. Our patient resided in Prince George's county in Maryland, an endemic region for lyme infection in the eastern USA, and it is suspected that infection occurred during frequent outdoor exposures while mowing his lawn near a wooded region. Normal laboratory values included C-reactive protein, erythrocyte sedimentation rate, uric acid, rheumatoid factor (RF), antinuclear (ANA) and citrulline antibodies, angiotensin converting enzyme, and human immunodeficiency virus (HIV) antibody. Following two weeks of doxycycline therapy for lyme infection and steroids for polyarthralgias, he was subsequently admitted with concurrent proximal muscle weakness of lower and then upper extremities, progressive dyspnea, facial and palmar dermatitis, and ten pound weight loss.

Physical examination of our patient revealed an ill appearing, afebrile male, with respirations of 25/minute and heart rate of 130/minute. Abnormal physical findings included bibasilar crackles without wheezing; violaceous skin eruption on the forehead, periorbital, and nasolabial folds; hyperpigmented papular palms with cracked, ulcerated fingertips; and decreased motor strength of 3/5 and 4/5 in the lower and upper extremities, respectively.

Laboratory evaluation included normal chemistry and blood counts, mildly elevated transaminases, ANA 1:40, positive anti JO-1 antibody, and negative cryoglobulins. Creatine phosphokinase (CPK) and aldolase levels were normal, which was suspected from recent corticosteroid therapy versus amyopathic dermatomyositis. Magnetic resonance imaging of the brain, lumbar spine and extremities were normal. Skin biopsies of the facial and finger lesions revealed advanced superficial and deep perivascular lymphocytic infiltrates, pigmented macrophages and perivascular lymphocytes in the superficial dermis with vacuolar degeneration and dermal mucin. The dermatopathologist felt that the clinicopathological findings most favored a diagnosis of dermatomyositis.

Evaluation for severe dypsnea was unrevealing including normal chest radiograph, nuclear stress testing, and echocardiogram. A working differential diagnosis revolved around the simultaneous occurrences of polyarthralgias and exuberant cutaneous and muscular symptoms, which could be related to his underlying HCV infection, an emerging connective tissue disease, or primary lyme infection itself. A muscle biopsy was contemplated to further evaluate myositis and presence of spirochetes. However, this was not completed due to our patient's severe debility and opinion that such a workup would not directly address the severe dyspnea, which was his most concerning and life-threatening condition at hand.

Repeat chest imaging confirmed bilateral ground glass opacities, diffuse interstitial infiltrates, and no pulmonary emboli on chest computed tomography scan. Our patient underwent aggressive therapy including steroidal, antimicrobial, bronchodilator therapy, and intubation (it was not completely clear the degrees to which his respiratory decompensation was attributable to worsening COPD exacerbation versus an interstitial pneumonitis, or pneumonia). His arterial blood glasses included pH 7.4, pCO _2 _40, PO _2 _104, CO _2 _27 96% on BiPap with 100% FIO2. The patient died one week later, and his family requested that an autopsy not be performed.

## Discussion

### Lyme disease

As the most common tick-borne infection in the USA, lyme disease manifests with clinical symptoms as early as one week to several months after bacterial infection [1]. Characteristic clinical manifestations include erythema migrans, cardiac and neuromuscular abnormalties, and arthralgias. The severity of these symptoms may vary due to complex interactions between the vector, bacteria, and host factors which ultimately result in inflammatory cascades (involving release of cytokines, chemokines, and other immune modulators, which inflict significant unintended host damage) [1]. Arthritis persists in ten percent of cases in spite of adequate antimicrobial therapy [1].

Nardelli *et al. *theorize that disparities in disease severities of lyme infection in different hosts may be understood in terms of genetic predispositions, immunologic differences, autoimmunity, and coexistent medical problems [1]. There is an interesting example of this complexity of interactions within a human with concurrent HCV and lyme infection such as in our patient. Byrnes *et al. *reported a male with chronic HCV infection, whose disease was eradicated following a life-threatening illness from co-infection with babesios, lyme borreliosis, and human granulocytic ehrlichiosis. They hypothesized that an exuberant cellular immune response from the infections led to eradication of HCV [2].

### Lyme disease diagnosis

Dermatomyositis has been described in the setting of lyme infection in only nine previous case reports [3-5]. Although lyme antibody testing is generally fraught with imprecise serological testing, either the pathognomonic EM rash or confirmatory western blot meets the prerequisites for the diagnosis of lyme infection per CDC criteria [6]. Since both enzyme-linked immunosorbent assay (ELISA) and western blot tests are indirect tests measuring the immune system response to infection rather than the bacteriologic agent itself, there are false positive results that may be seen for example in rheumatoid conditions [6]. False positive IgM antibody responses were eliminated in our case by normal ANA, DNA, and RF screens. Our case is unique because of the short time period between the time of diagnosis of acute lyme infection and the onset of aymopathic dermatomyositis and then severe and fatal pulmonary interstitial fibrosis.

### Dermatomyositis diagnosis

The classic diagnostic criteria for dermatomyositis described by Bohan included 1) skin lesions (heliotropic rash, gottron paules); 2) proximal muscle weakness of upper or lower extremities; 3) elevated CPK and 4) aldolase levels; 5) myalgias; 6) abnormal electromyography; 7) positive anti-Jo-1 antibody; 8) nondestructive arthralgias; 9) systemic inflammatory signs; and 10) Myositis [3-5]. Supporting studies include magnetic resonance imaging and autoantibodies to nuclear and cytoplasmic antigens.

**Figure 1:**
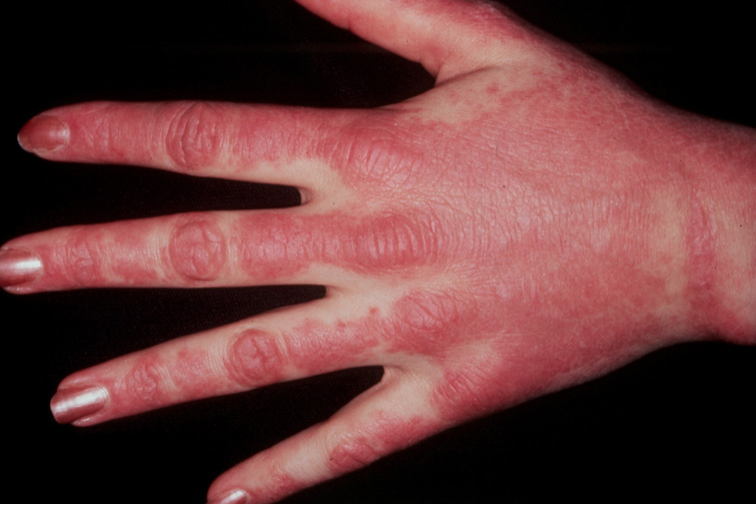
**Mechanic's hands in a patient with dermatomyositis**. Image reprinted with permission from emedicine.com, 2009. Available at: http://emedicine.medscape.com/article/1064945-overview

**Figure 2:**
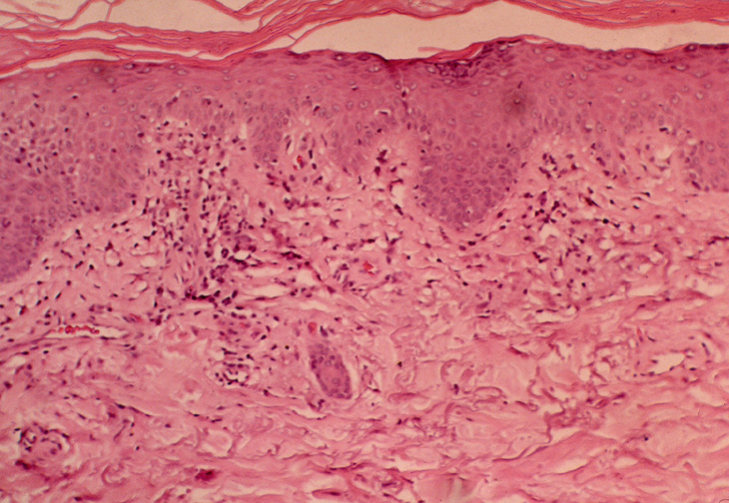
**Vacuolar changes of columnar epithelium and lymphocytic inflammatory infiltrates at the dermal-epidermal interface in dermatomyositis**. Image reprinted with permission from emedicine.com, 2009. Available at: http://emedicine.medscape.com/article/1064945-overview.

Other characteristic cutaneous findings include periungal erythema and telangiectasias; violaceous erythema and edema of the face in a photosensitive distribution; palmar panniculitis and hyperkeratosis (mechanic's hands) (Figure 1) which is associated interestingly with severe interstitial fibrosis as in our patient) [3-5]. Histological changes in dermatomyositis are likewise difficult to distinguish from those observed in lupus, including vacuolar changes of columnar epithelium, lymphocytic inflammatory infiltrates at the dermal-epidermal interface, and dermal mucin [3-5] (Figure 2).

### Lyme disease association with dermatomyositis

The association of lyme disease and dermatomyositis has been noted in only nine previous reports. In spite of a link of dermatomyositis with other varied medical conditions including infections, pregnancy, and medications (hydroxyurea and penicillamine), its pathogenesis remains elusive. Although our patient's medical conditions included both malignancy and hepatitis C infection, which are both associated with dermatomyositis, the temporal onset of dermatomyositis coincided with the time of diagnosis of acute lyme infection [7]. Steere *et al. *noted that perivascular lymphoid infiltrates in clinical myositis from lyme infection did not differ from polymyositis or dermatomyositis, suggesting systemic autoimmune damage perhaps through formation of autobodies cross-reacting with homologous host proteins in various organ systems [8]. Inflammatory plasma cells are prominent in early infection and induce a vascular thickening and collagen expansion [8].

Arniaud *et al. *described a case report of coexistent dermatomyositis, relapsing polychondritis, and positive lyme serology [5]. Most case reports have described dermatomyositis in the context of chronic lyme infection [3,5,9,10]. Dermatomyositis has been described in a forest owner with symptoms of dermatomyositis and positive polymerase chain reaction (PCR) testing for *B. burgdorferi *and detection of spirochete organisms in silver staining [9]. Also dermatomyositis has been diagnosed in an immunosupressed patient with seronegative lyme disease with positive anti Jo 1 autoantibodies and PCR testing for *B. burgdorferi *[10].

Less commonly, dermatomyositis has been described with acute lyme infection (Horowitz *et al.*), in similarity to our case presentation [4]. Our case similarly describes a short time course between time of diagnosis of acute lyme disease and onset of dermatomyositis. We speculate that our patient's other underlying medical problems (specifically malignancy and HCV infection), which may have acted synergistically with lyme infection to trigger dermatomyositis.

### Amyopathic dermatomyositis and interstitial lung disease

Amyopathic dermatomyositis is characterized by cutaneous manifestations, without myositis (normal CPK and aldolase levels). Euwer *et al. *first conceptualized that it may represent one continuum of disease spectrum with dermatomyositis-polymyositis on the other [11]. A failure to recognize amyopathic dermatomyositis early in the absence of myositis may cause considerable delays in diagnosis, which is relevant because amyopathic dermatomyositis may still be associated with life threatening systemic diseases including interstitial pulmonary fibrosis [12-14]. Our patient's presentation with sequential amyopathic dermatomyositis and severe pulmonary interstitial fibrosis is strengthened by the positive anti-JO1 antibody testing [13,14].

An alternative explanation to amyopathic dermatomyositis in this case, which is possible, is that early corticosteroid therapy for COPD stymied the emergence of clinical myositis. Interestingly, some patients with amyopathic dermatomyositis without clinical myositis still have abnormal findings on ultrasound, magnetic resonance imaging, or muscle biopsy [11,12]. In agreement with previous authors, we believe that overly strict interpretation of diagnostic criteria may lead to underdiagnosis and undertreament of amyopathic dermatomyositis in some patients who are not evaluated beyond clinical, immunological and enzymatic studies [11-14]. It is reasonable that given sufficient time, that progression of untreated lyme infection from acute to chronic stages may be associated with amyopathic dermatomyositis or full dermatomyositis-polymyositis [3-5].

Our patient's eventual diagnosis of interstitial pulmonary fibrosis was challenging because his respiratory complaints were initially largely attributed to COPD exacerbation. Our patient did not have a history of ILD and had normal chest radiographs on presentation, and ILD only ensued following development of the amyopathic dermatomyositis. However, early aggressive therapy with antimicrobials and corticosteroids (fortuitous treatment for both dermatomyositis and interstitial pulmonary fibrosis) was not successful in reversing outcome.

Although the association of lyme disease with dematomyositis, and link of dermatomyositis with interstitial lung disease are both described, to our knowledge this is the first case in which lyme disease is then linked to fatal ILD. Interestingly, in the medical literature, a case has been reported of lyme disease and respiratory decompensation through diaphgramatic paralysis [15]. Also, Silva *et al. *described three cases of acute respiratory failure from neuroborreliosis, and these patients had encephalopathy and brainstem abnormalities [16].

## Conclusions

**Figure 3:**
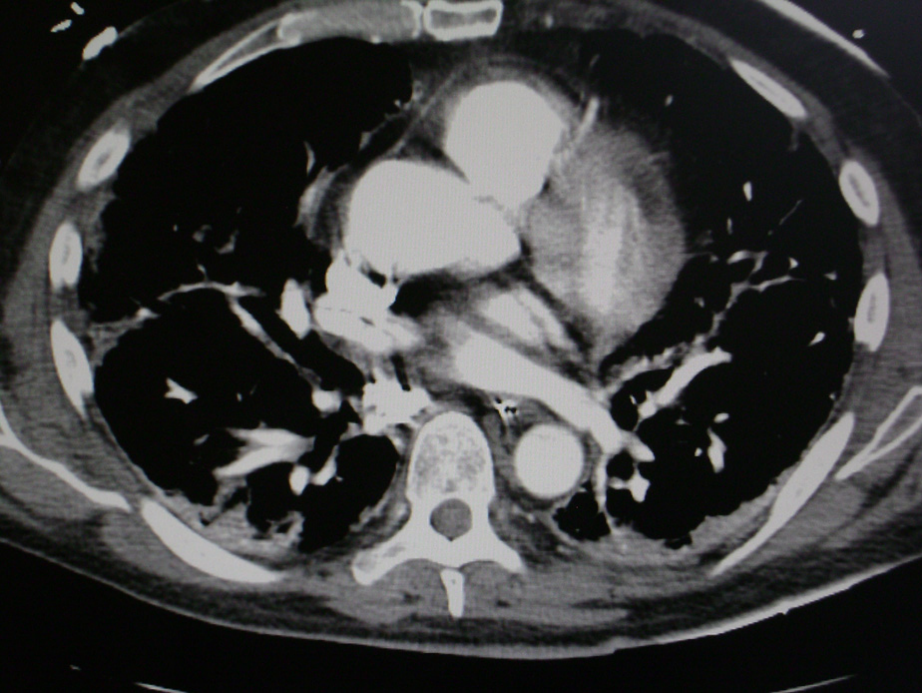
**Interstitial pulmonary fibrosis in our patient's computed tomography scan of the chest**.

Prognosis of dermatomyositis is related to severity of myopathy, related end organ damage, and coexistent malignancy [11,12]. Interstitial pulmonary fibrosis is perhaps the most pertinent and potentially life-threatening complication associated with amyopathic dermatomyosisits and yet its clinical features are not well understood [13,14] (Figure 3). Ideura *et al. *suggested that a subset of these patients are predisposed to rapid respiratory decompensation, and need to be identified early to enhance clinical outcomes [14].

To conclude, we describe a patient with acute lyme infection who presents with amyopathic dermatomyositis and rapidly progressive interstitial fibrosis. This case illustrates a potential for lyme disease to produce clinical syndromes and fatal complications that may be indistinguishable from those observed in primary connective tissue diseases. The corollary of this proposition is that in patients who are diagnosed with lyme infection who subsequently develop atypical muscular, respiratory or other systemic complaints, the possibility of severe rheumatological and pulmonary associations should be considered.

## Consent

Written informed consent was obtained from the patient's next-of-kin for publication of this case report and any accompanying images. A copy of the written consent is available for review by the Editor-in-Chief of this journal.

## Competing interests

The authors declare that they have no competing interests.

## Authors' contributions

HN, lead and corresponding author, managed this patient clinically, and helped to draft the manuscript and obtain the literature review. CL helped to draft the manuscript and obtain the literature review. HN helped to draft the manuscript and obtain the literature review. All authors have read and approved the final manuscript.
